# Operationalising Patient Engagement Through the Alberta Cancer Diagnosis Initiative: Recruitment Strategies for Diverse Populations in Health System Improvement

**DOI:** 10.1111/hex.70306

**Published:** 2025-06-05

**Authors:** Anna Pujadas Botey, Meaghan Brierley, Michelle Stiphout, Paula J. Robson

**Affiliations:** ^1^ Cancer Strategic Clinical Network, Alberta Health Services Calgary Canada; ^2^ School of Public Health University of Alberta Edmonton Canada; ^3^ Health Systems Knowledge & Evaluation, Alberta Health Services Calgary Canada; ^4^ Cancer Strategic Clinical Network, Alberta Health Services Edmonton Canada; ^5^ Cancer Research & Analytics, Cancer Care Alberta, Alberta Health Services Edmonton Canada

**Keywords:** health equity, health system improvement, patient engagement, quality improvement, recruitment strategies, service user involvement

## Abstract

**Background:**

Engaging diverse populations is critical for designing effective healthcare initiatives. However, strategies for recruiting participants to ensure meaningful engagement, particularly among harder‐to‐reach groups, remain underexplored. This study examines recruitment approaches used in the Alberta Cancer Diagnosis Initiative (ACDI) in Alberta, Canada, to address challenges in cancer diagnosis.

**Methods:**

A qualitative study, including seven interviews and four focus groups with 10 members of the ACDI project team (none of them patients or community members) and a review of 20 internal ACDI documents, was undertaken. Data were analysed using inductive coding, focusing on identifying recruitment strategies for engaging participants from diverse groups and considerations for facilitating recruitment.

**Results:**

Early commitment to diversity and relationship‐building informed recruitment strategies, including working with community brokers and health system navigators. Barriers included a limited time within the grant cycle to develop strong relationships with population groups. The team's capacity to learn from emerging issues, like intersectionality and language, was crucial to developing an adaptive approach to recruitment.

**Conclusions:**

Tailored and adaptive strategies, particularly broker‐ and community‐based approaches, are crucial for engaging diverse groups. Lessons learned can inform future initiatives seeking to involve these groups in healthcare decision‐making and programme development.

**Patient or Public Contribution:**

Patients and community members were actively involved in the ACDI during its planning and development. Their contributions informed engagement activities, ensuring the inclusion of diverse perspectives. This study examined the ACDI project team's perspectives on recruitment strategies and lessons learned, highlighting the importance of adaptive, community‐based approaches in engaging diverse participants.

**Abbreviations**
ACDI: Alberta Cancer Diagnosis Initiative
AHS: Alberta Health Services
AYA: adolescent and young adult
IWC: Indigenous Wellness Core

## 1 Introduction

Engaging populations affected by healthcare programming is crucial for the effective development of healthcare initiatives [1, 2]. The benefits of engaging diverse population groups are well‐documented [3, 4, 5], and several engagement strategies have been described in the literature [6, 7, 8, 9]. Much of the focus has been on the importance of engaging a diverse range of individuals, particularly those from vulnerable, marginalised or special‐needs groups, to ensure equitable healthcare coverage and decision‐making [10, 11]. There has also been emphasis on identifying ways to ensure that the voices of these groups are heard and included in decisions that address their unique needs [12, 13]. Additionally, challenges in accessing ‘harder‐to‐reach’ groups [11], tailoring processes to their needs [11] and managing the lengthy time commitments required for some engagement strategies are discussed extensively in the literature [3, 5, 14, 15, 16].

However, despite this wealth of knowledge on engagement, there remains a significant gap in the literature on recruitment strategies—the critical first step in enabling meaningful engagement, particularly among diverse and harder‐to‐reach groups [3, 5, 14, 15, 16]. This gap hinders our understanding of how healthcare delivery organisations and other stakeholders can effectively recruit these populations in the early stages of programme design, ensuring their voices are not only heard but acted upon. Addressing this gap is crucial for improving the effectiveness and inclusivity of healthcare programming, yet it remains unexplored [3, 4, 17, 18, 19].

In Alberta, Canada, the projected 56% increase in cancer cases by 2040 [20] highlights the urgency of addressing current challenges in cancer diagnosis. These challenges may include a lack of care coordination, unexplained variation in the length of time to diagnosis [21], and reliance on emergency departments and hospitals for diagnosis [22]. These issues contribute to poor experiences for both patients and providers, increased healthcare costs and potentially suboptimal patient outcomes [23, 24, 25, 26]. To tackle these issues and streamline cancer diagnosis in Alberta, the Alberta Health Services' (AHS) Cancer Strategic Clinical Network embarked upon the Alberta Cancer Diagnosis Initiative (ACDI) in 2021. The planning and development of ACDI involved engaging healthcare providers, patients and patient relatives who have experience with the cancer diagnosis process, as well as the broader diverse communities to which these patients belong. Understanding and addressing patient needs during cancer diagnosis was a key component of the programme planning and development process.

The ACDI provided an opportunity to study approaches taken by the project team in the real‐world design and application of strategies to recruit diverse groups of patients (and patient relatives) and community members to participate in engagement activities. Our aim was to address the existing gap in the literature on recruitment strategies used by members of healthcare delivery organisations who wish to ensure that the voices of diverse participant groups are heard and acted upon when designing initiatives to improve healthcare delivery. In this paper, we share the approaches and activities used and the lessons learned through the recruitment process. It contributes to the understanding of and considerations for recruiting diverse individuals to participate in and contribute to innovative health system improvement initiatives.

## 2 Methods

### 2.1 Data Sources and Collection

A document review was conducted to analyse internal ACDI materials supporting the planning, design and implementation of the initiative. The review covered 20 records, including the research proposal, project charter, project reports and activity tracking documents.

Additionally, focus groups and semi‐structured interviews were conducted via Zoom or Microsoft Teams with ACDI project team members involved in early‐stage ACDI planning and activities. These sessions explored team members' roles, experiences and reflections on recruitment and engagement efforts. Team members included both internal AHS personnel (employees or volunteers) and external consultants. While one volunteer was a patient/family partner, no patients or community members, in their capacity as such, were part of the ACDI project team or participated in the study.

We began with focus groups to facilitate discussion and exchange of ideas among team members, structuring them based on participant affiliation: two with internal AHS team members and two with external members. Each group participated in two sessions to allow for continued conversation and deeper exploration of emerging insights. All 13 team members were invited via email to participate, and all participated except three (two internal and one external) who were unable to join due to time constraints. Focus group sizes ranged from two to six participants. Following the focus groups, one‐on‐one interviews were conducted to deepen our understanding of individual perspectives and clarify emerging themes. In line with reflexive thematic analysis [27], data collection was guided by the goal of generating rich and meaningful insights rather than achieving thematic saturation. A total of seven interviews were conducted across all invited participants, some of whom required multiple sessions due to the breadth of topics covered and their availability.

The focus group and interview guides were developed by two researchers (M.B. and M.S.) based on related literature [8, 28, 29] and ACDI documents. While no formal pilot testing was conducted, the guides were iteratively refined during data collection, allowing for flexibility in exploring emerging insights. Topics covered are outlined in Appendix A.

To ensure validity and reliability, all interviews and focus groups were facilitated by M.B. and M.S., except in two instances when M.S. was unavailable. When both were present, they alternated by topic areas, with one facilitating and the other observing and taking field notes to maintain contextual details and adding follow‐up questions as needed. No non‐participants were present, and no participants withdrew. Focus groups lasted 60–90 min, while interviews lasted 45–60 min. All sessions were recorded, auto‐transcribed using Microsoft Teams and revised for accuracy (M.B.). While participants were not sent transcripts for review, they had opportunities to participate in multiple discussions, allowing them to build on previous insights. Data collection, including document review, focus groups and interviews, took place between November 2022 and January 2023.

All data collection activities were conducted by M.B. and M.S., both of whom have over 10 years of experience conducting health systems research and evaluation. Neither had prior relationships with participants, except that M.S. had collaborated with one participant on a separate study. As independent researchers, they approached this study with an interest in understanding recruitment strategies within the ACDI. Recognising the potential for researcher bias, they maintained a reflexive approach throughout data collection and analysis.

### 2.2 Analysis

Data analysis was supported by NVivo12 (QSR International, Australia) and conducted by the two researchers who collected the data (M.B. and M.S.). The process followed an iterative approach consisting of first‐cycle and second‐cycle coding [30]. In the first cycle, coding focused on familiarisation with the data, grouping text and identifying key themes. Through this process, an initial coding framework was developed and iteratively refined in ongoing discussions with the research team until a consensus was reached on areas for further exploration. The second‐cycle coding involved further refinement, synthesis and integration of insights, leading to a more nuanced understanding of the data. The interpretation of the identified codes provided evidence on the approaches and activities employed to recruit participants for engagement in the ACDI. Selected quotations illustrate the main findings.

### 2.3 Ethical Considerations

Explicit oral consent was obtained from interview and focus group participants. Participants were provided a copy of the consent form for their records. Ethical approval was granted from the Health Research Ethics Board of Alberta, Community Health Committee (Ethics ID# HREBA‐CHC‐21‐0054).

## 3 Results

Ten members of the ACDI project team participated, including six internal AHS staff (two managers, two research associates, one director and a postdoc fellow) and four external members (two engagement designers, one director and a patient/family partner). Participants included six women and four men, with ages ranging from 25 to 64 (two aged 25–34, four 35–44, two 45–54 and two 55–64). The analysis shed light on strategies employed by the ACDI project team to recruit participants from diverse patient and community groups who may have unique needs and concerns through cancer diagnosis, as well as considerations for facilitating this recruitment process.

### 3.1 Strategies for Participant Recruitment

The ACDI project team described approaching participant groups in ways to support connections and explored how people could, and would, best participate in the engagement activities. The recruitment process revealed the value of brokers, navigators and community relations.

#### 3.1.1 Brokers

Brokers are liaisons who help facilitate recruitment and engagement between their communities and healthcare teams. Embedded within specific groups such as older adults, newcomers or individuals experiencing homelessness or a precarious housing situation, brokers leveraged their established relationships to connect with individuals who may be difficult to reach through conventional means. They played a crucial role in overcoming barriers such as mistrust and language differences. Due to the project's tight timeline, the ACDI project team relied on brokers to leverage these connections rather than building them directly.

For instance, brokers working with older adults played a pivotal role in recruiting participants. Early attempts to recruit older adults directly involved outreach through local organisations and healthcare providers, distributing flyers and inviting individuals to participate. However, these efforts were met with limited success. This situation prompted the team to turn to patient advisory councils, who had established relationships and credibility within older adult communities, allowing them to tailor the recruitment process to meet these specific needs. Comprising older adult representatives, these councils not only facilitated recruitment but often participated themselves, offering valuable insights. ‘*I know I had brainstormed and all the organizations I listed for senior adults pretty much fell flat. And the way we ended up connecting with people in those groups was more through the patient advisory councils’* (I4).

Similarly, multicultural brokers working with newcomers helped overcome barriers such as language differences and mistrust in the healthcare system. Identified through organisations like the Low German Mennonite Society, Immigrant Outreach Society and African Cancer Support Group, these brokers ensured equitable participation and empowered communities to shape how engagement occurred. ‘*We ensured the selection was equitable and even in the actual engagement allowing the newcomer brokers to know that they actually have some power and they can actually direct how the engagement takes place and seeking their advice…’* (FG10).

In homeless and precariously housed communities, brokers were essential in protecting the vulnerable members of these groups. Rather than directly engaging with individuals in these communities, brokers stepped in as proxy data sources, ensuring the inclusion of their experiences without compromising their well‐being.

‘We did not engage directly with people who are unhoused or experiencing homelessness that we know of … some of the folks that we chatted with who were living rurally could have been couch surfing … so again, it's back to this kind of like the intersectional nature of what some of these challenges are.’

(FG2)

#### 3.1.2 Navigators

Adolescent and young adult (AYA) navigators are healthcare professionals embedded within the provincial cancer care system who work as part of multidisciplinary teams to support adolescents and young adults diagnosed with cancer. These navigators provided the ACDI project team with direct connections to participants already engaged with the healthcare system. The Cancer SCN's longstanding relationship with AYA navigators in Cancer Care Alberta facilitated recruitment through an ethics‐approved email to AYA patient panels. The ACDI project team also collaborated with the national organisation AYA Can (AYA Canada) to connect with their Alberta cohort.

‘Let's use AYA for example because we identified that as a gap. We found that expert person within AHS that had that advice and expertise and embedded them within the team so that we could go to them on a consultant basis and ask those questions right away, instead of flying blind and not having that expertise and just falling into it.’

(I13)

### 3.2 Community Relations

The Indigenous Wellness Core (IWC) is a provincial programme in AHS supporting Indigenous health needs and topics and focuses on building partnerships between AHS and Indigenous Communities. When contacted by the ACDI project team for advice on how to best engage with Indigenous Communities, the IWC brokered knowledge for the ACDI in distinct ways from the other broker examples in this project.

Drawing parallels with recruitment approaches used for homeless and precariously housed people, the IWC raised questions about engaging directly with Indigenous Community members. They emphasised the necessity of strong relationships and sufficient time to ensure meaningful engagement in the ACDI, deeming the existing timelines overly restrictive. Instead of substituting community engagement with mere data collection, the IWC referred the ACDI project team to a substantial body of research that had already been conducted. Consequently, rather than advocating for and facilitating new engagements with Indigenous Communities for the ACDI, they recommended reviewing previous engagements and existing knowledge, complemented by a document review of published works involving the community.

‘We didn't actually complete the Indigenous engagement piece, and I think that was OK, because if we had tried to do it without the advice on how to do it, we wouldn't have been successful. So now we've been able to just start to bend the curve a little bit on opening that door for us to continue the engagement with those populations.’

(FG10)

In summary, ACDI project team members shared that brokers, navigators and community relations facilitated recruitment and helped regulate access to community members, ensuring protection from potential exploitation, avoiding unnecessary or repetitive engagement and safeguarding the integrity of the process. These facilitators highlighted the importance of building trust and relationships, which requires time and effort beyond tight timelines and funding cycles. As one participant noted, ‘*I don't know that anyone ever wrote that down, but the timelines were too tight’* (I4). Another highlighted the need to recognise and build upon prior research and engagement efforts:

‘A lot of research had already been done, so I think there was a good example inside of that around saying that we sometimes say we want to engage and maybe really we need to spend time understanding what's already been done.’

(I4)

Brokers, navigators and community relations allowed the ACDI to test possibilities, learn and adjust to new ways of connecting with participant groups when appropriate.

## 4 Recruitment Considerations

Several elements were identified that supported the recruitment and early engagement of the participant groups. These elements included recognising intersectionality, language, time, relationships, flexibility and a willingness to learn.

### 4.1 Intersectionality

The recruitment process initially involved targeting specific population groups and categorising participants into distinct groups. However, the ACDI project team soon realised that participants often belonged to more than one group, which allowed them to contribute insights beyond a single categorisation or context:

‘… it wasn't as cut and dry as “I'm in this group.” “I'm in that group.” You know, things overlapped; people were more intertwined in that way … often the way people describe themselves is more informative than the checkboxes that we want to put them in. And so that “lenses” piece really just became the way we started our conversations or engagement. And so, it was really just an opportunity for people to describe who they were.’

(I4)

Multiple lenses also ‘overlapped*’*: ‘*Not everybody just wore one hat. In fact, most people wore multiple hats’* (I5). Interview and focus group participants further elaborated on the complexity of participants' identities with specific examples:

‘There's always that overlap. So, for example, when I say overlap, we have older adults that live in rural communities. We have immigrants that are caregivers to their parents, who are also immigrants, older adults and then we have maybe immigrants that also belong to the LGBTQ+ sexual and gender minority groups. So, it's never … a person doesn't belong to a single subgroup.’

(I7)

### 4.2 Language

Language emerged as a consideration in recruitment, particularly when involving newcomer and immigrant communities. The ACDI project team enlisted the support of brokers proficient in the languages spoken by these communities. Some brokers were able to communicate directly with participants in their native languages, facilitating more effective engagement. In instances where communication in English was feasible, project staff led the interactions. For sessions requiring translation, brokers assisted with translating materials or interactions, ensuring accuracy and clarity. This dual approach, supported by tailored facilitation guides, allowed for inclusive and accessible engagement with both newcomers and the broader community. ‘*And I think it was a little bit different the way I approached brokers than the way like [Name 2] did because I didn't have the language barrier’* (I4).

The recognition of language was a significant factor underscoring the ACDI project team's commitment to overcoming communication barriers from the beginning and ensuring that the voices of all participants were heard and valued in the engagement process.

### 4.3 Time and Relationships

Building meaningful relationships with potential participant groups took time. A noted challenge was the lack of time to build relationships before inviting individuals to participate in the engagement activities.

‘I think one of the challenges would be that it did take us longer than we anticipated to forge those relationships. And so having to adhere to the timelines, I would love to have seen this extend over the summer because it was so rich.’

(FG6)

Examples of the importance of the personal connection provided by the ACDI project team with brokers and navigators also emerged as important for identifying diverse participants who could provide commentary on the cancer diagnosis experience.

### 4.4 Flexibility and Willingness to Learn

Flexibility and openness to learning were crucial to recruitment. Letting go of pre‐conceived ideas and actions resulted in one participant noting they ‘*course corrected an awful lot’* (FG10) even at the stages of participant identification and recruitment. Ethics amendments, decisions to shift approaches to connect with new communities, exploring whether people in these communities would like to participate and deciding to revisit which communities to engage in the moment were all part of the iterative nature of the early stages of the ACDI. The willingness to learn also came from the ACDI project team and others involved in the ACDI, making the active decision to learn through these processes. ‘*Please remember, this is how we have been thinking about the work from inside the health system, and it will have blind spots. You might see it differently and we would be excited to learn from you.’* (ACDI Sample Facilitation Guide).

## 5 Discussion

Engaging with diverse populations is crucial for the planning and development of effective healthcare programmes [3, 4, 5, 31]. To ensure meaningful engagement and gather valuable insights, it is essential to hear from all programme users, not just those easily reachable [31, 32, 33]. While good intentions often underpin efforts to engage diverse groups, translating these intentions into meaningful action in real‐world settings requires adaptive strategies, adequate resources and a willingness to learn. The experiences of the ACDI project team illustrate the complexities of recruitment, demonstrating that successfully engaging diverse populations necessitates more than just outreach. It calls for thoughtful, flexible approaches tailored to the specific needs of each group.

The findings from this study highlight the necessity of dedicating sufficient time and resources to foster connections and understand what works for whom and in what particular contexts. Consistent with existing literature [11], the ACDI project team recognised that engagement efforts must be responsive to the distinct circumstances of different communities. Several factors were identified that can help mitigate challenges early on, ensuring more effective engagement. These include using brokers to overcome language barriers [34, 35, 36], maintaining openness and responsiveness to participants' feedback [11, 34] and leveraging existing relationships through brokers and navigators [3, 34, 35, 37, 38].

The ACDI project team's experience aligns with Massie and Boothe's [11] call for reflexivity in recruitment approaches. Recognising early on that their strategies needed flexibility, the ACDI project team embraced the idea of ‘turning recruitment on its head’ when necessary. Rather than rigidly adhering to initial plans, they focused on listening, learning and adapting strategies continuously to meet the specific needs of targeted groups. For instance, instead of directly engaging with Indigenous and homeless or precariously housed populations, the team shifted to a strategy informed by brokers. These brokers advised against rushing engagement without first establishing trust and relationships, reinforcing that successful recruitment often depends on prior rapport‐building. In some cases, this also meant recognising that building on existing knowledge about a community may be more ethical than repeatedly requesting their participation in research. This shift highlights the importance of prioritising trust‐building over achieving recruitment goals in a short period of time.

A key finding from this study is the recognition of the complexity of intersectionality in participant recruitment [34, 39]. Intersectionality—where individuals belong to multiple overlapping identity groups—is increasingly seen as a significant challenge in patient engagement efforts, especially when aiming to represent diverse groups [40, 41, 42, 43]. Initially, the ACDI project team attempted to categorise participants into distinct groups, but they soon realised that many individuals straddled multiple identities, such as age, rurality, race and gender. This realisation aligns with the growing recognition that intersectionality is crucial for achieving meaningful patient engagement [34, 39]. The team adapted their recruitment strategies to better address the needs of participants with intersecting identities, with brokers playing a key role in leveraging their established relationships with participant groups to facilitate tailored recruitment. This approach proved essential in fostering inclusive engagement.

The recognition of intersectionality also led the ACDI project team to conclude that a one‐size‐fits‐all approach was ineffective for recruitment. Tailored strategies were necessary to meet the unique needs of individuals with intersecting identities. Future research should examine how intersecting identities influence engagement and participation outcomes, as individuals with multiple overlapping identities face barriers that traditional recruitment models may not adequately address. Research evaluating tailored engagement approaches—such as trauma‐informed, intersectional frameworks and culturally adaptive recruitment strategies—could provide actionable insights for improving inclusivity. Additionally, future studies could explore the long‐term impact of integrating diversity, equity and intersectionality training in the early stages of project planning, complemented by the development of novel methods for assessing how these efforts translate into sustained engagement. Addressing these gaps is critical for developing scalable, evidence‐based frameworks that enhance engagement across diverse communities. The importance of relationship‐building was another critical insight from the ACDI project team's recruitment efforts, aligning with existing literature [3, 43, 44]. Effective relationship‐building requires significant time and effort. Of relevance, the ACDI project team's approach underscored the need to prioritise quality relationships over transactional engagements aimed solely at data collection. This is particularly important when engaging groups with negative past experiences with research or evaluation, or those who may have felt tokenised or exploited in previous efforts [5, 45, 46, 47, 48]. Brokers and navigators played a pivotal role in building trust and facilitating connections with participants. Sometimes described as cultural or community brokers in the literature [34, 35], brokers in our study acted as intermediaries who addressed language barriers and served as valuable data sources and support systems for participants.

By leveraging these existing relationships, the team could better adapt to the unique needs of diverse participant groups, an approach well supported by the literature, emphasising trust and relationship‐building in community engagement [3, 8, 9, 34, 44]. However, the role of brokers varied significantly. Some brokers actively worked to identify and connect with potential participants, while others served as advisors, helping the team develop relationship‐building strategies tailored to specific communities. This variation in brokers' involvement underscores the importance of recognising that not all brokers are the same, and their level of engagement depends on the scope of the project and their unique relationships with the community. These valuable lessons reinforce the need to explore the feasibility and potential impact of brokers' roles early in the recruitment process to ensure effective and inclusive engagement strategies.

Sharing the experiences, challenges and lessons learned while recruiting diverse individuals and communities for the ACDI aims to advance real‐world engagement practices. Emphasising continuous evaluation and adaptation is crucial to improving engagement efforts. Ongoing research into the time needed for relationship‐building, along with continuous knowledge‐sharing across diverse settings, is vital for fostering meaningful engagement. By continually learning and revising approaches based on insights gained, we can develop strategies that effectively meet the needs of all service users, ultimately promoting their meaningful participation in health system planning and development.

### 5.1 Limitations

The findings of this study should be considered in light of some limitations. First, due to staff transition, ACDI project team members who left the team before this study were not included in the data collection process. As a result, the findings may not fully reflect all team members' perspectives. Additionally, as suggested in the literature, the reliance on virtual interviews and focus groups, while necessary due to limited resources for in‐person interactions and the convenience of online formats, may have restricted the ability to capture nonverbal cues, potentially impacting the flow of conversation and reducing spontaneity in responses [49, 50]. However, given that the ACDI project team primarily worked in virtual formats throughout the project, participants were accustomed to this mode of communication, which may have mitigated some of the limitations associated with virtual interactions. Furthermore, due to time and resource constraints, perspectives from those recruited and those assisting in recruitment, such as brokers and navigators, and community members, were not included in the study. Their inclusion could have enriched the findings by providing a broader, more diverse set of views, thus reducing the potential for self‐reporting bias from ACDI project team members. This gap highlights the need for further research into participant identification and recruitment to deepen our understanding of effective engagement strategies for people from diverse populations invited to inform improvements to healthcare delivery.

## 6 Conclusion

This examination of the recruitment strategies used in the ACDI provides novel insights into recruiting diverse communities to inform health systems programming and highlights crucial, yet underexplored, considerations for large healthcare systems seeking to engage patients and community members in co‐designing initiatives to improve healthcare delivery. A central takeaway from this study is the importance of broker conversations, navigator support and community partnerships in recruiting diverse populations. Our findings also emphasise the importance of relationship‐building and flexible engagement strategies in overcoming participation barriers. These insights hold significant potential for shaping future healthcare initiatives and policies, ensuring they are more inclusive and responsive to diverse patient needs. While this study focuses on Alberta, Canada, the lessons learned are relevant to other healthcare settings aiming to improve recruitment and engagement with diverse populations. This study not only addresses a gap in the literature but also serves as a call to action for healthcare systems to adopt more inclusive, flexible and tailored recruitment strategies with adequate time for relationship building. Future research should continue to explore these approaches, furthering our understanding of how to best engage diverse participant groups in healthcare planning and delivery, with a particular focus on including the perspectives of the people being recruited.

## Author Contributions

**Anna Pujadas Botey:** conceptualisation, formal analysis, investigation, methodology, supervision, project administration, writing – review and editing, writing – original draft. **Meaghan Brierley:** conceptualisation, data curation, formal analysis, investigation, methodology, writing – original draft, writing – review and editing. **Michelle Stiphout:** conceptualisation, data curation, formal analysis, investigation, methodology, project administration, writing – original draft, writing – review and editing. **Paula J. Robson:** conceptualisation, writing – review and editing.

## Ethics Statement

Ethical approval was granted from the Health Research Ethics Board of Alberta, Community Health Committee (Ethics ID# HREBA‐CHC‐21‐0054).

## Consent

Explicit oral consent was obtained from interview and focus group participants.

## Conflicts of Interest

The authors declare no conflicts of interest.

## Supporting information

Appendix A.

## Data Availability

The data that support the findings of this study are available on request from the corresponding author. The data are not publicly available due to privacy or ethical restrictions.
