# Supplementary material for: Operationalising Patient Engagement Through the Alberta Cancer Diagnosis Initiative: Recruitment Strategies for Diverse Populations in Health System Improvement
Source: Health Expect. 2025 Jun 5;28(3):e70306. doi: 10.1111/hex.70306 (PMC12141090; doi:10.1111/hex.70306)
Supplement: Supplementary file 1 — Appendix A. [file HEX-28-e70306-s001.docx]

**Appendix A: Interview and focus group guide**

**Introductory questions (participant information)**

1. Please introduce yourself and then describe your role and how you became involved in the Alberta Cancer Diagnosis Program Engagement. How were you invited to participate?
2. What prompted you to participate/join?

**Reach (who participated in the process, and were they the right people?)**

We want to know more about your impressions of the engagement and the people who participated.

1. Describe the process of your engagement.
2. What did/does your role/experience/background bring to this goal?
   1. How do you think your (beliefs, values, and attitudes) affected your input about the program?
3. In your opinion, how important is the Alberta Cancer Diagnosis (ACD) Program relative to other initiatives? Do you think other people feel the same?
4. Who else do you think would have been valuable to include in this process?
5. How was the facilitation of your engagement/session? Did you sense everyone had the opportunity to speak? To what extent were the participatory processes embraced and used to make improvements in the Alberta Cancer Diagnosis Program?

**Effectiveness (to what extent did the process achieve its objective?)**

We want to know more about how the engagement process clarified the problems ACD is addressing.

1. Explain how you arrived at the blueprint? (use the remaining questions as prompts)
2. How did these components contribute?
   1. System dynamics map?
   2. System dynamics map colours?
   3. Barrier and enabler loops (appraisal loop barrier, help seeking loop barrier, diagnostic loop barrier, pre-diagnosis enabler)?
   4. Design principles (e.g., seeing the whole person, building relationships, enabling choice and access?
   5. Imagine ifs
3. How does the blueprint translate into practical change? (what’s next? So what?)
   1. What are you doing with this plan?
4. What kinds of impacts has the engagement had? Probe: Can you provide an example?

**Implementation (to what extent was the process implemented as intended?)**

We know that there are some subtle things about different places/facilities that can influence how engagements are conducted, so we have a few questions about that topic.

1. Have you gone back to the Albertans involved:
   1. Do they see what they said reflected in the blueprint?
   2. Does the blueprint address the challenges they reported?
2. What do you think would help implement the evaluation findings from your perspective?
3. What kinds of supports do you think would help sustain the program? Why would it make a difference?
   1. Probes: examples of supports [program leads, wider organization]? Are there any changes you would recommend to make the program more successful?

**Closing question**

1. Are there any other comments or thoughts about the Alberta Cancer Diagnosis (ACD) Program Engagement that you would like to share?
